# Investigation of standardized training of radiation oncology residents for gynaecological tumours in China

**DOI:** 10.1186/s12909-023-04264-7

**Published:** 2023-05-02

**Authors:** Shuai Sun, Ke Hu, Fuquan Zhang, Xiaorong Hou

**Affiliations:** Department of Radiotherapy, Peking Union Medical College Hospital，Chinese Academy of Medical Sciences & Peking Union Medical College, Beijing, 100730 China

**Keywords:** Radiation oncology, Residents, Standardized training, Gynaecological malignant tumour

## Abstract

**Background:**

Radiotherapy standardized training (ST) has been conducted for 7 years in China. This investigation evaluated the difficulties of and need for ST of radiation oncology residents (RORs) for gynaecological tumours (GYN) in China.

**Methods:**

An anonymous online survey was conducted on the “Questionnaire Star” platform. The questionnaire contained 30 questions, including the basic information of the students, their knowledge of radiotherapy theory, training on GYN, the difficulties and needs they faced, and possible solutions.

**Results:**

A total of 469 valid questionnaires were collected, resulting in a valid response rate of 85.3%. During the ST, only 58–60% of RORs received training in GYN, with a median clinical rotation time of 2–3 months. Among the RORs surveyed, 50.1% knew the physical characteristics of brachytherapy (BRT), and 49.2% could choose the appropriate BRT for patients. At the end of ST, 75.3% were able to complete the target delineation in GYN independently, and 56% were able to complete the BRT operation independently. The scarcity of GYN patients, insufficient teaching awareness of superior doctors, and lack of interest are the main reasons why ST cannot meet the standard.

**Conclusion:**

In China, the ST of RORs in GYN should be strengthened, the teaching awareness of specialist trainers should be increased, and the curriculum should be optimized, especially the curriculum for specialist operation and a strict assessment system.

## Background

Radiotherapy is the main treatment for common gynaecological malignant tumours such as cervical cancer. A combination of external beam irradiation and brachytherapy is needed in treatment, which involves high requirements for the theoretical knowledge of radiation oncology, radiation biology and physics and the clinical skills of radiation oncologists. However, most medical schools in China do not offer courses in radiation oncology, which makes it difficult for newly graduated residents to master it in the short term.

Currently, the training programme of 5 + 3 + X is mostly adopted for radiation oncology residents (RORs) in China; it involves 5 years of undergraduate medical study, 3 years of standardized training (ST), and X years of specialized training (generally 2–4 years). In 2014, radiation oncology (RO) was included in the national ST of residents for a period of 3 years. The main clinical rotation departments currently include the radiotherapy department for 10 months (head and neck tumour, chest tumour, abdominal tumour, gynaecological tumour, and others), the general internal medicine department for 10 months (cardiology department, respiratory department, digestive department, infection department, neurology department, emergency department, and ICU), and tumour-related departments for 13 months (otorhinolaryngology department for 1 month, stomatology department for 1 month, imaging department for 2 months, pathology department for 2 months, internal oncology department for 3 months, and tumour surgery/general surgery for 4 months). At the end of ST, clinical theory and practical ability are assessed with a combination of the daily comprehensive score. Follow-up specialized training has not yet entered the formal implementation stage, but most hospitals have a specialized training mode. According to the requirements of ST, the training time for gynaecological tumours (GYN) is approximately 2 months, but the minimum number of cases is 10. The requirements indicate the need to master the target delineation of GYN and brachytherapy (BRT) operations.

In the current ST mode, we conducted a questionnaire survey on radiotherapy ST in GYN to clarify the difficulties and needs related to ST and to explore a better training model.

## Methods

The online anonymous survey was conducted with the help of the “Questionnaire Star” platform, and each participant was limited to completing the survey once. The survey was conducted from January 27, 2022, to February 16, 2022. The respondents were residents specializing in radiotherapy in China. The questionnaire included 30 questions, including the basic information of the students, their knowledge of radiotherapy theory, their training in gynaecological tumours, the difficulties and needs they faced, and possible solutions.

The collected data was analyzed using SPSS 19.0 software, and the comparison of variable composition ratios was carried out using the chi-square test.

## Results

### Basic information

**Fig. 1:**
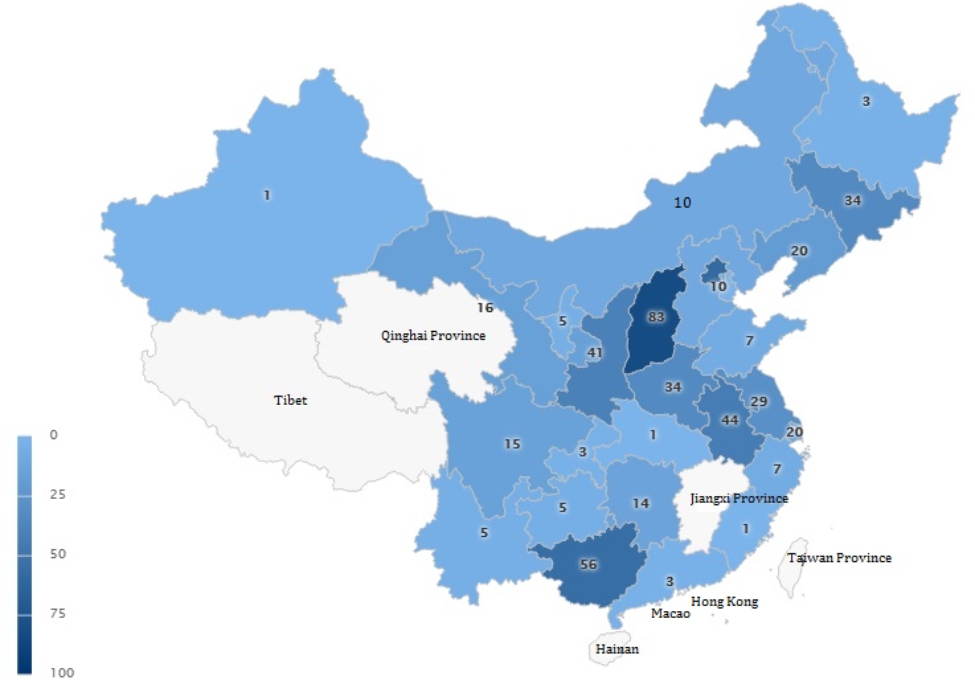
The geographical distribution of the participants in this survey

A total of 550 questionnaires were distributed, out of which 528 were received. Fifty-nine respondents who were not residents were excluded, including 15 chief doctors, 41 deputy chief doctors, 1 chief technician, and 2 technicians. Finally, 469 valid questionnaires (85.3%) were analyzed. The collected questionnaires were from 27 provinces, autonomous regions, and municipalities across China. However, there were no data from Qinghai Province, Jiangxi Province, Hainan, Tibet, Taiwan Province, Hong Kong, and Macau. The specific distribution is shown in Fig. 1.

Of the respondents, 417 (88.9%) were from tertiary hospitals and 52 (11.1%) were from second-level hospitals. Among them, 392 (83.6%) were from teaching hospitals. Among the hospitals where the RORs worked, 96.4% carried out CT simulation positioning, 25.8% carried out MRI simulation positioning, 95.5% carried out intensity modulated radiotherapy, and 70.6% carried out BRT.

### Current status of ST of RORs in GYN

Among the 469 RORs, 407 (86.8%) received ST on RO and 62 residents received non-RO ST，including 4 (0.9%) who received ST in obstetrics and gynecology, 43 (9.2%) who received ST in internal medicine, and 15 (3.2%) who received ST in surgery.

Among all RORs, 327 (69.7%) junior physicians who had been engaged in radiotherapy for 1–3 years received ST on RO and 142 (30.3%) senior physicians who had been engaged in radiotherapy for 4–6 years completed ST on RO. In the two groups, only 192 (58.7%) and 84 (59.2%) underwent GYN rotation in ST (P = 0.506 ,χ^2^ = 0.046), respectively; 79.2% had a rotation period of 1 to 6 months, and half of them had a rotation period of 2 to 3 months.

Among the 469 residents, the number of GYN patients they treated is shown in Table 1. For junior doctors, the number of patients treated with definitive radiotherapy for cervical cancer was mostly 11–20 cases, and the number of patients treated with postoperative radiotherapy for GYN was 1–5 cases. Only 320 (68.2%) residents had performed gynaecological examinations for patients.

**Table 1:** Number of GYN patients treated by RORs

Number of cases	Definitive radiotherapy for cervical cancer		Postoperative radiotherapy of GYN	
	Junior physician	Senior physician	Junior physician	Senior physician
0	12.8%	1.4%	12.5%	1.4%
1–5	15.0%	4.2%	22.0%	7.7%
6–10	16.5%	4.2%	20.5%	7.0%
11–20	19.0%	10.6%	18.0%	11.3%
21–30	12.8%	9.9%	10.1%	9.9%
31–40	6.4%	13.4%	5.2%	13.4%
41–50	1.2%	1.4%	1.5%	6.3%
> 50	16.2%	54.9%	10.1%	43.0%
χ^2^	99.04		108.19	
*P*	<0.001		<0.001	

Among 430 RORs who had treated GYN patients, 176 (40.9%) had experience in applicator implantation for patients with GYN. Among them, 48 (27.3%) thought they were skilful at BRT, 81 (46.0%) were familiar with BRT, 34 (19.3%) were generally familiar with BRT, and 13 (7.4%) were not familiar with BRT.

**Fig. 2:**
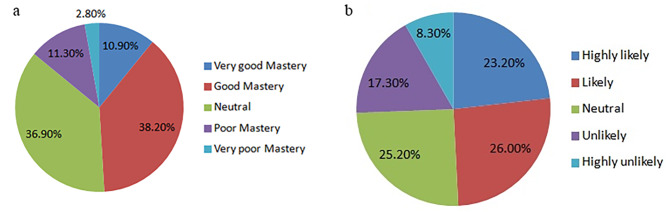
Residents’ knowledge of physical characteristics (2a) and indications for BRT (2b)

Among all participants, 92.1% thought that BRT was very important for patients with locally advanced cervical cancer, 7.7% thought BRT was important, 0.2% thought BRT was generally important, and no participants thought BRT was not important. Only 50.1% of RORs knew the physical characteristics of BRT, and 49.2% thought they could choose the appropriate BRT for patients, as shown in Fig. 2a and b.

At the end of the current ST, only 75.3% of RORs can independently complete the target delineation of GYN patients, and 56% of RORs can independently complete the BRT.

### Difficulties and needs

#### Difficulties

**Fig. 3:**
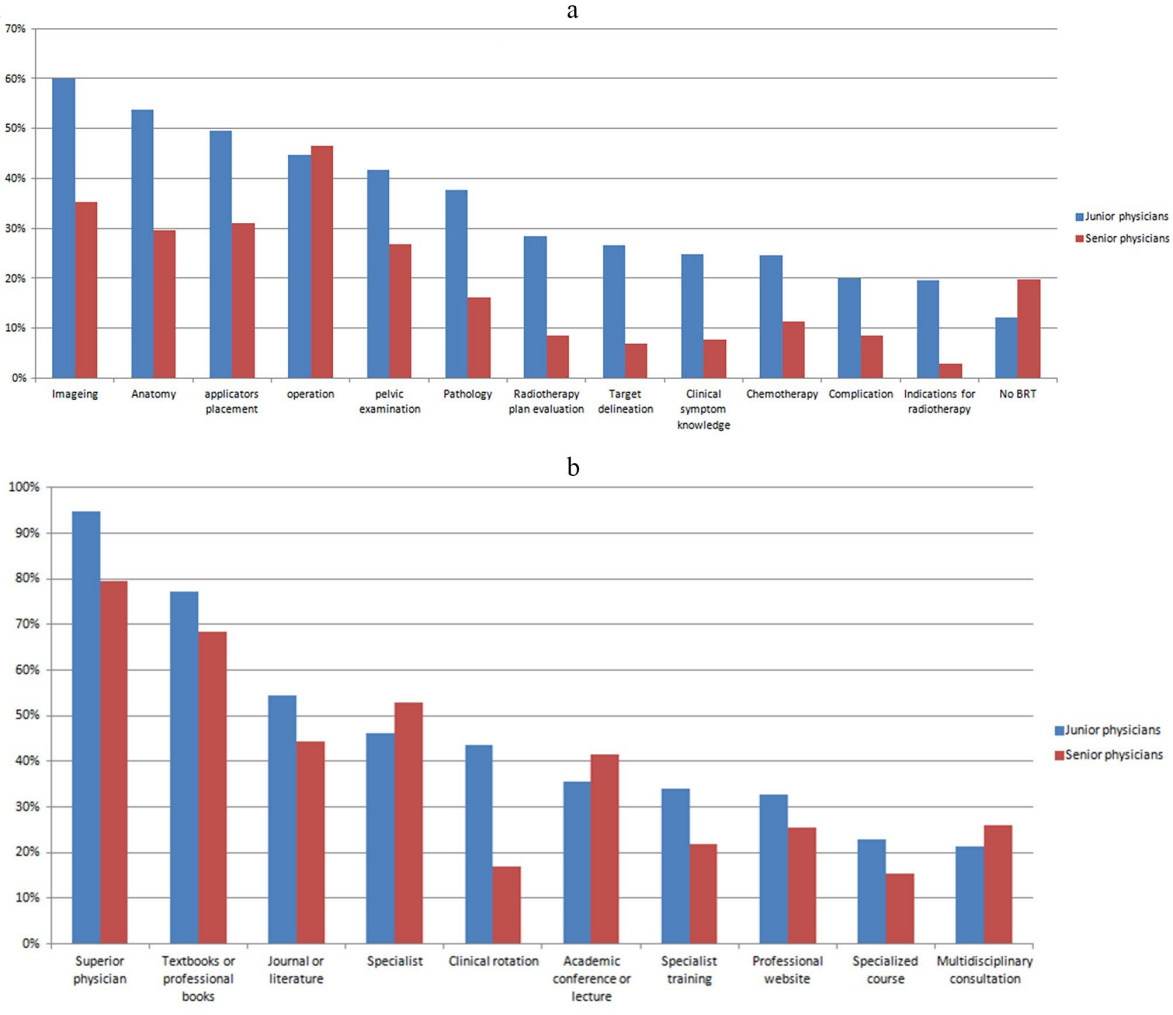
Difference in problems encountered (3a) and approach to solving problems (3b) in treating GYN patients between junior and senior residents

The problems faced by junior and senior RORs in treating patients with GYN are different, as shown in Fig. 3a. With regard to the mastery of basic knowledge and skills, senior physicians were significantly better than junior physicians (P < 0.05), but there was no significant difference in the understanding of surgical procedures between the two groups (*P* = 0.714, χ^2^ = 0.134).

In practical clinical work, when residents encounter problems, junior physicians usually prefer to consult their superiors, textbooks or professional books, periodicals, or literature to solve the problems. The proportion of junior physicians who consulted their superiors was significantly higher than that of senior doctors (94.8% vs. 79.6%, *P* = 0.000), as shown in Fig. 3b.

At the end of the ST in GYN, some residents do not reach the level of treating GYN patients independently. The survey showed that the main reasons include the scarcity of GYN patients, insufficient teaching awareness of superior physicians, and personal lack of interest, accounting for 65.7%, 50.3% and 19%, respectively.

#### Requirements

The survey results showed that at the end of the ST, to achieve the ability to treat GYN patients and complete BRT independently, the minimum number of patients to be treated was 11–20 patients with definitive radiotherapy for cervical cancer and 11–20 patients with postoperative radiotherapy.

**Fig. 4:**
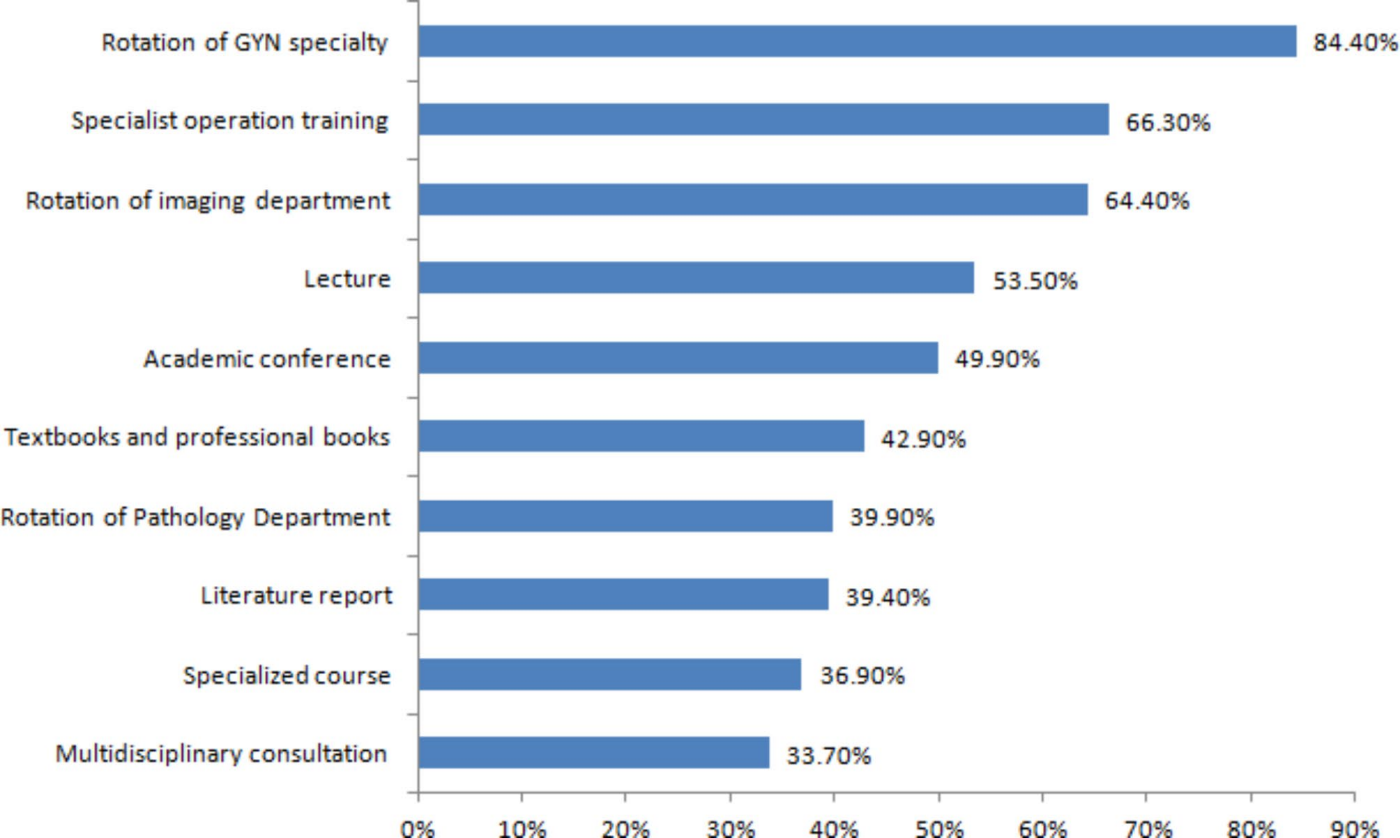
The additional training programme expected in the ST of RORs for GYN

In addition, in the ST, RORs who intend to engage in the specialty of GYN in radiotherapy oncology in the future can increase the time of GYN training, participate in specialist operation training and increase their rotation in the imaging department. See Fig. 4 for other specific requirements.

All surveyed residents agreed to set up a formal course of brachytherapy, of whom 86.6% agreed to include an assessment of brachytherapy competence at the end of the training.

## Discussion

The ST of residents plays an important role in postgraduation medical education. Improving the post competency of resident physicians is the core of ST for resident physicians.

Radiotherapy ST has been carried out for 7 years in China. However, according to the survey results, the proportion of residents who received GYN training in the ST was only maintained at approximately 60%, which means that not all RORs receive GYN training in the ST. Even if they received GYN training, the median rotation time could only be maintained at 2–3 months.

During the actual training of the GYN subspecialty in radiation oncology, the survey results showed that the number of cases treated by RORs basically met the requirements of ST but could not meet the clinical needs of trainees. In addition, due to the lack of basic theory and skill training, although most residents treated patients with GYN and almost all believed that BRT was important for GYN, only 3/4 of them could delineate the target volume independently and half of them could implant applicators for patients independently with knowledge of the physical characteristics and indications of BRT.

There are three main reasons for failing to achieve the goal of ST. First, the number of patients was insufficient. Second, the superior physicians’ awareness of teaching was not strong. Third, the current training model cannot stimulate learners’ interest in GYN radiotherapy.

Most of the RORs hoped to increase the rotation of the GYN specialty, increase professional operation training, set up formal BRT courses, and increase professional operation skill assessment to improve the effect of ST.

Current radiotherapy techniques for GYN mainly include external irradiation and brachytherapy. Training in external irradiation technology is feasible because it is not limited by equipment. However, brachytherapy training is the opposite. In recent years, several surveys of residents in America, Europe and Australia have shown that approximately 40–70% of residents have insufficient BRT training during their residency training due to the number of patients, limited equipment, and other reasons [1–5].

In China, according to the latest cancer data, cervical cancer ranks fifth (11.34/100,000) in the incidence of female malignant tumours, uterine cancer ranks eighth (6.64/100,000), and cervical cancer ranks seventh in the mortality rate (3.36/100,000). The incidence and death from cervical cancer are still on the rise [6]. Therefore, gynaecological malignant tumours remain among the main diseases affecting women’s health in China. The distribution of patients may be uneven, but the overall number of patients is large. Increasing the flow of resident training between hospitals can compensate for the lack of training caused by the lack of patients during training.

RORs still prefer the help of superior doctors to solve clinical problems, so the teaching awareness of superior doctors needs to be improved. In daily teaching, multiple forms of teaching modes should be combined [4, 5, 7, 8] to increase students’ interest in GYN.

The setting of specialized courses is a common requirement in the ST of RORs, such as imaging, radiation physics, and biology [9]. Brachytherapy is indispensable for the training of the GYN subspecialty in radiotherapy. However, because it involves patient-specific operation, improper operation during BRT will produce uncomfortable experiences for patients and reduce the operator’s confidence. In the event of an error, the teacher may terminate the resident’s operation to avoid irreparable harm to the patient. Therefore, the training process will be affected. The simulation course of BRT can help trainees repeat operations infinite times through mechanical models or virtual reality simulation to increase their proficiency and skills in operation, establish their confidence and improve their post competence [10, 11]. At the same time, rotation in the imaging and gynaecological oncology departments will meet the training needs of most residents.

This survey was aimed at China’s radiation oncology residents, with widely distributed data sources and a valid questionnaire response rate of 85.3%, making the conclusions generally applicable nationwide. However, there were some limitations to this survey. Since the main objective was to investigate the standardized training mode in gynecological tumor subspecialties for radiation oncology residents in China, it is unfortunate that no detailed investigation was conducted on brachytherapy. In the future, we plan to investigate on the relevant details of brachytherapy.

## Conclusions

The ST of the gynaecological oncology subspecialty of radiation oncology in China has not been fully popularized, and residents’ mastery of specialized skills and theories still cannot meet the training requirements. It is necessary to increase the teaching awareness of specialized training teachers and continue to optimize the curriculum, especially by improving the curriculum for specialized operation and strict assessment systems.

## Data Availability

The datasets used and/or analysed during the current study are available from the corresponding author on reasonable request.

## Abbreviations

ST: standardized training
RORs: radiation oncology residents
GYN: gynaecological tumours
BRT: brachytherapy
RO: radiation oncology
